# Factors Associated with Patient Interest in a Digital Exercise and Mind–Body Program for Patients Undergoing Active Cancer Treatment: A Secondary Analysis of a Randomized Clinical Trial

**DOI:** 10.3390/cancers18193092

**Published:** 2026-09-23

**Authors:** Karolina Bryl, Esin Namoglu, Susan Chimonas, Raymond E. Baser, Hyowoun Jyung, Jun J. Mao, Angela K. Green

**Affiliations:** 1 Integrative Medicine and Wellness Service, Department of Medicine, Memorial Sloan Kettering Cancer Center, New York, NY 10065, USA; 2 George Washington University School of Medicine and Health Sciences, Washington, DC 20037, USA; 3 Department of Epidemiology & Biostatistics, Memorial Sloan Kettering Cancer Center, New York, NY 10065, USA; 4 Gynecologic Oncology Service, Department of Medicine, Memorial Sloan Kettering Cancer Center, New York, NY 10065, USA

**Keywords:** exercise, mind–body therapies, digital health, patient interest, cancer, cancer treatment, integrative medicine

## Abstract

**Background/Objectives**: As digital health interventions gain traction in oncology care, understanding factors influencing patient participation is critical to optimizing delivery and enhancing symptom management. Sociodemographic and clinical factors associated with patient interest in a 12-week digital exercise and mind–body program among patients undergoing cancer treatment were examined. **Methods**: A secondary analysis of the IMPROVE trial was conducted. This trial evaluated the effects of Integrative Medicine at Home (IM@Home) versus enhanced usual care on fatigue among adults with breast, thoracic, gynecologic, or head/neck cancers or melanoma. Analyses were limited to participants randomized to the IM@Home arm (*N* = 99). Patient interest was operationalized as the number and type of class registrations. Associations with sociodemographic and clinical characteristics were examined using chi-square or Fisher’s exact tests and Wilcoxon rank-sum tests. **Results**: Among 99 participants (mean age 60.8 ± 12.7 years; 91.9% female), 88.9% registered for at least one class and 70.7% registered for ≥3 classes. Yoga (64.6%) and fitness (63.6%) were the most commonly selected modalities. Receipt of radiation therapy was associated with fewer class registrations (*p* = 0.01). Older participants were more likely to register for music therapy (*p* = 0.01), non-Hispanic participants were less likely to register for tai chi/qigong (*p* = 0.04), and participants who were not employed were less likely to register for yoga (*p* = 0.02) but more likely to register for dance (*p* = 0.01). **Conclusions**: Select patient-level characteristics were associated with preference for different modalities in a digital integrative medicine program, underscoring the importance of flexible, patient-centered design to support personalization, equity, and engagement in supportive oncology care.

## Simple Summary

People receiving cancer treatment often experience symptoms such as fatigue, anxiety, and reduced quality of life. Although integrative medicine therapies, including exercise and mind–body approaches, can help manage these symptoms, little is known about which therapies patients are most interested in while they are receiving treatment. This study examined patient preferences for a 12-week online program offering exercise and mind–body classes, including yoga, fitness, meditation, tai chi, music therapy, and dance. Most participants registered for at least one class, and nearly three quarters registered for three or more classes. Preferences for specific class types differed according to age, ethnicity, employment status, cancer stage, and treatment type. These findings highlight the importance of offering patients a choice of evidence-based supportive care options that better meet their diverse needs and preferences. Such flexible, personalized programs have the potential to improve access, engagement, and symptom management for people undergoing cancer treatment.

## 1. Introduction

Individuals undergoing systemic cancer treatment or radiation therapy often experience substantial symptom burden, including fatigue, anxiety, depression, and insomnia, which impair quality of life, daily functioning, and treatment adherence [1,2]. Cancer and its treatment can contribute to systemic inflammation and metabolic dysregulation, with inflammatory cytokines and adipose-derived signaling factors potentially contributing to tumor progression and treatment-related symptom burden, including fatigue [3,4]. These symptoms frequently co-occur and are associated with increased emergency department visits, unplanned hospitalizations, and poorer clinical outcomes, particularly among patients with advanced disease [5,6]. Effective symptom management during treatment is therefore recognized as a cornerstone of high-quality, patient-centered oncology care [7]. Integrative medicine modalities such as mind–body therapies (e.g., yoga, tai chi, meditation) and structured exercise programs are effective approaches to reduce treatment-related symptoms [8,9,10,11] and promote physical and emotional well-being [10,11,12,13]. Reflecting this evidence, practice guidelines from major oncology societies now recommend these therapies for symptom management [13,14,15].

However, persistent disparities in use of integrative medicine therapies remain which limits their broader implementation into clinical practice. For example, research shows integrative medicine usage rates among non-Hispanic whites are nearly double those of Hispanic and Black adults [16], and women are also more likely than men to utilize these services [17,18]. Yet, few studies have examined which patients are interested in specific integrative medicine modalities or whether modality selection is associated with sociodemographic or clinical characteristics. Moreover, most evaluations focus on patients who have completed treatment, and far less is known about how patients intend to engage with integrative medicine during active systemic therapy when symptom burden is high and competing demands may limit participation [19,20]. Understanding what types of integrative medicine modalities are of interest to different patients in active treatment could help clinicians match therapies to both clinical needs and personal preferences, therefore maximizing therapeutic benefit [21,22].

To explore these critical issues, we conducted a secondary analysis of the IMPROVE randomized clinical trial among participants randomized to the Integrative Medicine at Home (IM@Home) intervention, a 12-week digitally delivered mind–body and exercise program for patients experiencing fatigue and undergoing systemic or radiation treatment. We examined sociodemographic and clinical characteristics associated with the number and type of classes for which participants initially registered. Understanding factors associated with early registration is essential to promoting intervention relevance, motivational readiness, and care personalization among patients with cancer.

## 2. Materials and Methods

### 2.1. Study Setting

The study was conducted at Memorial Sloan Kettering (MSK) Cancer Center, a large, urban academic comprehensive cancer center in New York City, NY, USA.

### 2.2. Study Design

The present study is a secondary exploratory analysis of data from the IMPROVE trial, a single-center pragmatic, single-blind, two-arm, parallel-group patient-reported outcomes (PRO)-basket RCT evaluating a digital IM@Home versus an enhanced usual care (EUC) control among patients with eligible solid tumors from predefined cancer groups on active treatment who reported moderate or greater fatigue over the past week as measured by One-Item Fatigue Screen (OIFS) [23,24]. Interventions were administered over 12 weeks, and PRO measures were assessed at baseline and weekly thereafter for 12 weeks. The study was conducted in accordance with the Declaration of Helsinki, approved by the Institutional Review Board at MSK (#21-369), and registered at Clinicaltrials.gov (NCT05053230). Full details of the trial design and primary results have been published elsewhere [25]. All participants provided written informed consent, including consent for the use of research data in secondary analyses. This present study only focused on participants who were randomized to the IM@Home group (*N* = 99), who had access to the IM@Home program and its range of digital integrative medicine modalities, to examine sociodemographic and clinical characteristics associated with initial class registration and modality selection.

### 2.3. Participants and Procedures

Eligible participants were English-speaking adult patients diagnosed with breast, thoracic, gynecologic, or head/neck cancers or melanoma actively receiving systemic or radiation treatments experiencing moderate or greater fatigue (rated 4 or higher on the 0–10 OIFS numerical rating scale [23,24]) over the past week. Patients were also required to have a score ≥ 60 on the Karnofsky Performance Status (KPS) scale [26] and have a life expectancy of over 6 months. Patients were ineligible if they had evidence of cognitive impairment or were participating in concurrent integrative medicine trials.

Trained research staff conducted an initial screening with patients to confirm their study eligibility. Eligible patients provided informed consent and completed baseline assessments. Patients were then randomly assigned to receive IM@Home or EUC in a 1:1 ratio using permuted block randomization stratified by tumor type with a secure computer system that ensured full allocation concealment. The study statistician and outcome assessors were masked to treatment assignment. All study investigators including the primary investigators, co-investigators, outcome assessors, and statisticians were blinded to treatment assignments. Participants, research coordinators, and mind–body and exercise instructors were not blinded. This secondary analysis was further limited to participants randomized to IM@Home arm.

#### Integrative Medicine at Home (IM@Home)

The IM@Home program offered 23 live, Zoom-based mind–body and exercise classes per week. The classes ranged from movement-based classes (e.g., fitness, dance) to mind–body classes (e.g., yoga, tai chi) to mind-focused classes (e.g., meditation, music therapy). Each class was from 30 to 60 min. Upon randomization, a clinical research coordinator contacted participants by phone to introduce the IM@Home program, provide an overview of the classes, and assist with class registration. Participants could register for any number and combination of classes, and study staff recorded all selections. Detailed program description has been published elsewhere [23].

### 2.4. Outcomes

Class registration was defined as the number and types of IM@Home classes selected by each participant at the study start. Class registration was used as a proxy for patient interest, as both the number and type of classes selected provide an observable measure of participants’ initial willingness to engage with and prioritize specific integrative medicine modalities during active treatment. Importantly, registration does not equate to class attendance, participation, or adherence. These represent distinct stages of intervention engagement that require separate measurement. The 23 weekly classes were grouped into six mutually exclusive categories: fitness (*N* = 9), yoga (*N* = 4), tai chi/qigong (*N* = 4), meditation (*N* = 3), music therapy (*N* = 2), and dance (*N* = 2). Because the number of weekly offerings differed across modalities, registration frequency reflects both participant selection and the availability of classes within each modality. Therefore, registration counts were interpreted descriptively and were not considered direct measures of relative modality preference. For analysis, participants were categorized as either low registration (0–2 classes) or high registration (3 or more classes), consistent with prior research [25,27]. This threshold also aligns with the American Cancer Society’s recommendation of at least 150 min of moderate-intensity activity per week, ideally spread across three days [28], and represents a guideline-concordant and clinically meaningful benchmark for examining participation in digital mind–body and exercise interventions.

### 2.5. Exposures

Sociodemographic variables (age, gender, race, ethnicity, marital status, education, employment) were collected at baseline via patients’ self-report.

Clinical variables (cancer type, disease stage at diagnosis, cancer treatment modality, KPS score) were extracted at enrollment from MSK’s electronic health record (EHR).

Patient-reported symptoms (fatigue, anxiety, depression, distress, insomnia) were collected at baseline via MSK Engage, a secure platform for collecting and visualizing patient-generated health data integrated within the MSK’s Patient Portal. Fatigue severity was measured by the Brief Fatigue Inventory (BFI) a reliable and valid measure of cancer-related fatigue (Cronbach’s α, 0.82 to 0.97) [29]. The BFI contains 3 fatigue severity items and 6 fatigue interference items scored on a Likert scale from 0–10, where 1–3 indicates “mild” fatigue severity/interference; 4–6, “moderate”; and 7–10, “severe”. Average BFI severity score is obtained by averaging all three fatigue severity items. Anxiety and depression were measured by the Hospital Anxiety and Depression Scale (HADS) a 14-item tool with 7 items each for depression and anxiety subscales, as scored on a 4-point Likert scale (0–3) [30]. Symptom distress was measured by the Edmonton Symptom Assessment System (ESAS), a validated tool assessing the intensity of 10 symptoms (pain, fatigue, nausea, depression, anxiety, drowsiness, shortness of breath, appetite, feelings of well-being, and sleep, on an 11-point Likert scale (0–10 per item). The ESAS scores are categorized into the physical score (0–60; pain, fatigue, nausea, drowsiness, dyspnea, appetite), emotional score (0–20; anxiety and depression), and total symptom distress score (0–90; physical symptoms, emotional symptoms, and well-being), with higher scores indicating greater symptom burden [31]. In this study, the total symptom distress score was used. Insomnia severity was measured by the Insomnia Severity Index (ISI) a 7-item tool with each rated on a 5-point Likert response scale (e.g., 0 = no problem; 4 = very severe problem), yielding a total score ranging from 0 to 28 with higher scores representing more severe insomnia symptoms [32].

### 2.6. Data Analysis

Sociodemographic, clinical, and class registration data were summarized using descriptive statistics, and reported as frequencies and percentages for categorical data and medians with interquartile ranges (IQR) for continuous variables. Sociodemographic variables were dichotomized for analysis as younger (<65 years) vs. older (≥65 years); white vs. non-white; non-Hispanic or Latino vs. other; married, living w/partner vs. single, divorced, separated, widowed; and employed vs. unemployed (including retirees). Cancer treatment modalities (chemotherapy, radiation, immunotherapy, targeted therapy) were analyzed as separate binary variables (yes vs. no), and patients could receive multiple treatments concurrently. Class registration was examined both descriptively and inferentially, overall and by class type. Baseline symptom scores were categorized into binary outcomes: BFI severity mild/moderate (1–6) vs. severe (≥7); HADS-A/D no (<8) vs. yes (≥8); ESAS mild (10-<40) vs. moderate/severe (≥40–90); and ISI no/mild (0–14) vs. moderate/severe (≥15). Chi-square tests were used to explore associations between sociodemographic and clinical variables (e.g., age, cancer stage, symptoms) and class registration (binary 0–2 vs. ≥3 classes), as well as class types. Wilcoxon rank-sum tests and Kruskal–Wallis tests were used to test associations between the sociodemographic/clinical variables and the number of registered classes. Participants with missing data for a specific variable were excluded from analyses involving that variable. Given the exploratory nature of this secondary analysis and the modest sample size, multivariable modeling was not performed because of concerns regarding limited statistical power and potential model overfitting. Accordingly, observed associations should be interpreted as exploratory and may be subject to unmeasured confounding. All analyses were performed in R version 4.2.2., with a significance level set at α = 0.05.

## 3. Results

### 3.1. Study Participants

As previously reported, 609 patients were screened between October 2021 and March 2023. A total of 200 were enrolled in the parent study and randomized to either IM@Home (*N* = 99) or EUC (*N* = 101) [25]. All IM@Home participants (*N* = 99) were included in this secondary analysis. Among them, the mean age was 60.8 ± 12.7 years. The majority were female (91.9%), white (78.8%), non-Hispanic or Latino (88.9%), married or living with a partner (62.6%), and had a college education or above (69.7%) (Table 1).

### 3.2. Class Registration

Of 99 participants in the study, a total of 88 (88.9%) registered for at least one class, while 11 (11.1%) did not register for any classes. Overall, yoga (*N* = 64, 64.6%) and fitness (*N* = 63, 63.6%) were the most frequently selected classes, followed by meditation (*N* = 49, 49.5%), tai chi/qigong (*N* = 47, 47.5%), music therapy (*N* = 17, 17.2%) and dance (*N* = 14, 14.1%) (Table 2). Among participants who registered for at least one class (*N* = 88, 88.9%), the median number of class types registered was 3 (range 1–6), with 6.8% registering for all available class types. The majority of participants (*N* = 70, 70.7%) registered for ≥3 classes with the most popular class selections among this participant subgroup being fitness (*N* = 56, 80.0%), yoga (*N* = 53, 75.7%), meditation (*N* = 43, 61.4%), and tai chi (*N* = 41, 58.6%). Among those who registered for 1 or 2 classes (*N* = 18, 18.2%), yoga was the most common class chosen (*N* = 11, 61.1%), followed by fitness (*N* = 7, 38.9%), meditation (*N* = 6, 33.3%), and tai chi (*N* = 6, 33.3%).

### 3.3. Sociodemographic and Clinical Factors Affecting Number of Class Registrations

White patients were more likely to register for at least one class than non-white patients (93.6% vs. 71.4%, *p* = 0.04). Patients undergoing radiation were also less likely to register for any class compared with those not receiving radiation (80.9% vs. 96.2%, *p* = 0.02). No sociodemographic characteristics were associated with the number of classes registered. Radiation therapy was the only clinical factor associated with fewer class registrations (median 3 [IQR, 2.25–3.75] vs. 4 [IQR, 3.00–5.00], *p* = 0.01) (Table 1).

### 3.4. Sociodemographic and Clinical Factors Affecting Type of Class Registrations

Older participants were more likely to register for music therapy than younger participants (*p* = 0.01), non-Hispanic or Latino participants less likely to register for tai chi or qigong than other participants (*p* = 0.04), and those who were not currently employed were less likely to register for yoga (*p* = 0.02) but more likely to register for dance (*p* = 0.01) than those who were employed.

Of nine clinical characteristics, three were associated with type of class registration. Specifically, those with stage 0-III disease at diagnosis were more likely to register for dance than those with stage IV (*p* = 0.03), and patients with melanoma were more likely than other cancer types to select music therapy (*p* = 0.04) (Table 2). Cancer treatment modality, fatigue severity, performance status (KPS), ESAS score, anxiety, depression and insomnia had no association with type of class registration.

## 4. Discussion

This secondary analysis of the IMPROVE trial characterized initial class registration among participants randomized to the IM@Home intervention and examined sociodemographic and clinical characteristics associated with registration and modality selection. Among IM@Home participants, most registered for at least one class, and several patient-level characteristics were associated with registration preferences or selection of different modalities. The findings provide insight into factors influencing initial interest and class type selection during active cancer treatment that may help inform future development and implementation of flexible, patient-centered digital supportive-care program design. Our findings emphasize the clinical value of offering accessible digital programs with a range of evidence-based modalities that accommodate treatment-related demands and align offerings with patients’ needs and preferences to enhance participation and integration into routine oncology care.

In this study, among participants who were offered the IM@Home program, nearly nine in ten participants registered for at least one IM@Home class, and the majority registered for three or more classes, indicating a high level of initial interest in digital integrative medicine therapies during the demanding period of active treatment. Within our predominantly non-Hispanic white female sample, sociodemographic characteristics were not associated with the number of classes registered among those who enrolled in at least one class. While these findings contrast with prior research reporting lower integrative medicine interest among patients with less education or older age, and variable engagement by race and ethnicity [33,34,35,36], they should be interpreted cautiously given the sample’s homogeneity and limited statistical power for subgroup comparisons. Notably, race was associated with registering for any class, but not with the number of classes selected, suggesting that factors influencing initial interest may differ from those shaping depth of engagement. However, given the small subgroup sizes and sample homogeneity, this finding should be interpreted cautiously. In univariate analyses, the only clinical factor significantly associated with fewer class registrations was receipt of radiation therapy; however, this association was not statistically significant after adjustment for age, race, and employment status. Although not directly assessed in this study, the observed pattern may reflect demanding radiation therapy schedules and treatment-related fatigue or toxicity that may limit patients’ interest in engaging with additional health-promoting activities even in digital formats [37,38,39]. Future research should examine how treatment demands, social determinants, and cultural factors influence interest in digital integrative medicine programs, particularly among more diverse and underrepresented populations, to inform equitable and scalable models of supportive care delivery. Furthermore, qualitative interviews with participants who registered for classes and those who did not could provide additional insight into factors influencing initial registration, modality selection, and barriers to engagement.

Fitness and yoga were the most frequently registered modalities in this predominantly female and non-Hispanic white sample. These classes also had more weekly offerings available than other modalities, and registration patterns may therefore reflect both class availability and participants’ familiarity with, perceived health benefits of, and widespread recognition as part of supportive oncology care [28,40,41]. Prior studies show that patients often wish to remain physically active during treatment and view exercise as an empowering and restorative, even when fatigued or deconditioned [42,43]. Yoga, in particular, is well-tolerated during chemotherapy and associated with improvements in fatigue, sleep, and mood [44,45]. Our findings suggest that yoga and fitness may serve as entry points for supportive care programming, offering familiar, adaptable formats that address a range of physical and psychological needs. However, given our predominantly non-Hispanic white female sample, these patterns may differ among male patients or more diverse cohorts, underscoring the need for broader representation in future research.

Meditation and tai chi/qigong, though less frequently selected, may be perceived as lower-intensity and more accessible during active treatment. While associations with baseline fatigue severity or symptom burden were not observed, prior literature suggests these modalities may be particularly appealing to patients with limited physical capacity or higher symptom burden. As gentle, low-impact modalities that emphasize internal awareness and stress regulation, they may be appealing to individuals with anxiety, limited energy, or reduced physical capacity, patients who are often underserved by traditional exercise-based interventions [43,46]. During active treatment, when patients’ physical and psychological reserves are constrained, such modalities may feel more manageable and sustainable than high-intensity exercise [47]. This again highlights the importance of offering a range of options that align with patients’ evolving needs and treatment stages.

Music therapy and dance, although the least selected overall, were nonetheless chosen by approximately one in six participants (music therapy) and one in seven participants (dance). While their lower registration frequency may reflect class availability as well as less familiarity or perceived relevance in a clinical context, their selection suggests they offer distinct benefits. For instance, older adults, who often report loneliness, were more likely to register for music therapy, a non-strenuous group-based modality shown to support social connection, reduce anxiety, improve mood, and enhance quality of life [48,49]. Indeed, research indicates that older patients often gravitate toward more contemplative or expressive therapies over more physically demanding interventions [48,49]. In univariate analyses, patients who were unemployed or retired, who often face social isolation, were more likely to register for dance (a modality that fosters joyful emotional expression and social connection [50,51,52]), while employed participants were more likely to register for yoga; these associations may reflect differences in schedule availability, perceived physical demands, or other factors associated with employment status. One possible explanation is that yoga may appeal to employed individuals as a convenient modality for stress reduction and recovery from sedentary work [53]. These findings underscore the role referring clinicians and research staff can play in educating patients about the evidence base and therapeutic benefits of less familiar integrative therapies, such as music therapy or dance. Proactive education about the purpose, accessibility, and potential impact of these modalities may expand patient interest and participation. The registration patterns observed among older and unemployed patients highlight the value of offering a broad array of interventions to meet varied interests and needs.

Together, these findings suggest that even during active systemic treatment, patients selected a range of integrative modalities for initial registration when offered flexibility and choice across different cancer types and stages. In contrast to single-modality interventions, offering a variety of evidence-based options may provide patients with greater opportunity to select modalities that align with their individual circumstances and interests [21,54]. However, class registration should be distinguished from subsequent participation, engagement, and adherence. In the present study, registration was used as a proxy for initial interest, whereas subsequent stages of engagement were outside the scope of this secondary analysis. While patients in this sample most frequently registered for yoga and fitness, relying on these modalities alone may inadvertently exclude patients who are not interested in or do not feel capable of participating in them. Including less conventional options such as tai chi, music therapy, and dance, supports personalization and inclusivity. Beyond offering a range of modalities, culturally responsive programming and patient education may be important to ensure that available options are perceived as relevant and accessible across diverse populations. Although not assessed in this study, the ability to self-select modalities may enhance relevance and autonomy, and may contribute to subsequent engagement [55]. These findings support consideration of flexible, diverse, and accessible options tailored to individual needs and preferences in integrative oncology. Further research is necessary to understand how integrative oncology can meet the needs of men and other races and ethnicities given low representation in the current study.

Several limitations should be noted. First, the modest sample size limits statistical power, particularly within smaller demographic, clinical, and cancer-type subgroups. The restriction to participants randomized to IM@Home who self-selected to enroll in the parent trial also limits generalizability to the broader oncology population. Underrepresentation of racial, ethnic, and tumor-type subgroups, exclusion of non-English-speaking individuals, small cancer-type subgroups (e.g., melanoma; *N* = 4), and low male representation (*N* = 8; 8.1%) further limit generalizability. Second, this was a single-site study conducted at a major academic cancer center, which may limit applicability to other care settings. Third, participants who enroll in clinical trials may differ from the broader oncology population in motivation, technology access, or willingness to engage with supportive-care interventions. Although technology access did not emerge as a barrier during parent trial recruitment, device access, internet connectivity, digital literacy, prior experience with online programs, and caregiver support may be important barriers in under-resourced populations. Fourth, analyses were based on class registration rather than attendance, and we did not capture changes in preferences over time. Lastly, because multiple sociodemographic and clinical characteristics were examined across registration outcomes and individual modalities, the possibility of type I error due to multiple comparisons cannot be excluded. The use of univariable analyses also limits the ability to account for potential confounding among sociodemographic and clinical characteristics. Therefore, findings should be considered exploratory and require confirmation in larger, adequately powered studies. Future studies should intentionally recruit more diverse populations, assess digital access and literacy directly, and examine class attendance, engagement, and symptom response over time to better understand the relationship between patient needs, preferences, and outcomes. Integration of patient-reported outcomes and preference-based algorithms into digital platforms may enhance alignment of therapeutic modalities and support care personalization.

## 5. Conclusions

This secondary exploratory analysis identified sociodemographic and clinical characteristics associated with initial class registration and modality selection among participants randomized to a digitally delivered mind–body and exercise program during active cancer treatment. The findings suggest that offering a range of evidence-based modalities may provide patients with opportunities to select supportive-care options that align with their individual circumstances and interests. The high level of interest observed among IM@Home participants supports the clinical potential of multi-modal, accessible, digital integrative medicine programs as a strategy to address symptom burden and improve quality of life in oncology populations. Future research should focus on optimizing delivery and expanding access for underrepresented populations to maximize engagement and clinical benefit. Validation in larger, diverse oncology populations is needed before these findings can inform broadly applicable digital-program personalization strategies and support their integration into broader supportive cancer care.

## Figures and Tables

**Table 1:** Participant Sociodemographic and Clinical Characteristics Associated with Class Registration and Number of Classes Selected.

	Total (*N* = 99)	Registered Yes ^1^		Number of Classes Registered ^2^		Registered for 3 or More Classes ^3^	
	*N* (%)	*N* (%)	*p*-Value *	Median (IRQ)	*p*-Value **	*N* (%)	*p*-Value *
**Gender**							
Female	91 (91.9)	80 (87.9)	0.30	3.00 (3.00–4.00)	0.44	63 (69.2)	0.28
Male	8 (8.1)	8 (100.0)		3.00 (2.00–5.00)		7 (87.5)	
**Age (years)**							
<65	57 (57.6)	51 (89.5)	0.83	3.00 (3.00–4.25)	0.41	38 (66.7)	0.30
≥65	42 (42.4)	37 (88.1)		3.00 (3.00–5.00)		32 (76.2)	
**Race**							
White	78 (78.8)	73 (93.6)	**0.04**	3.00 (3.00–4.50)	0.60	58 (74.4)	0.12
Non-White	21 (21.2)	15 (71.4)		3.00 (0.00–5.00)		12 (57.1)	
**Ethnicity**							
Non-Hispanic or Latino	88 (88.9)	80 (90.9)	0.07	3.00 (3.00–4.00)	0.74	63 (71.6)	0.59
Other	11 (11.1)	8 (72.7)		5.00 (3.00–6.00)		7 (63.6)	
**Marital Status**							
Married, living w/partner	62 (62.6)	58 (93.5)	0.33	3.00 (3.00–4.50)	0.72	47 (75.8)	0.37
Single, divorced, separated, widowed	27 (27.3)	22 (81.5)		4.00 (3.00–5.00)		18 (66.7)	
**Education level**							
High school or some college	20 (20.2)	20 (100.0)	0.09	3.00 (2.00–4.00)	0.60	15 (75.0)	0.82
College degree or higher	69 (69.7)	60 (88.2)		3.00 (3.00–5.00)		50 (72.5)	
**Employment**							
Employed	46 (46.5)	40 (87.0)	0.34	3.00 (2.25–4.00)	0.37	31 (67.4)	0.21
Not employed	43 (43.4)	40 (93.0)		3.00 (3.00–5.00)		34 (79.1)	
**Cancer Type**							
Breast	35 (35.4)	29 (82.9)	0.22	3.00 (2.00–4.00)	0.24	22 (62.9)	0.44
Gyn	22 (22.2)	20 (90.9)		3.00 (2.50–4.00)		16 (72.7)	
Head/Neck	13 (13.1)	11 (84.6)		3.00 (2.00–4.00)		8 (61.5)	
Thoracic	25 (25.3)	24 (96.0)		4.00 (3.00–5.00)		21 (84.0)	
Melanoma	4 (4.0)	4 (100.0)		4.00 (2.50–4.75)		3 (75.0)	
**Cancer Stage**							
Stage 0–3	67 (67.7)	58 (86.6)	0.29	3.00 (3.00–5.00)	0.48	49 (73.1)	0.44
Stage 4	32 (32.3)	30 (93.8)		3.00 (2.00–4.00)		21 (65.6)	
Radiation ***							
Yes ^a^	47 (47.5)	38 (80.9)	**0.02**	3.00 (2.25–3.75)	**0.01**	30 (63.8)	0.23
No	52 (52.5)	50 (96.2)		4.00 (3.00–5.00)		40 (76.9)	
Chemotherapy ***							
Yes	37 (37.4)	33 (86.8)	0.94	3.00 (2.00–4.00)	0.20	25 (67.6)	0.76
No	62 (62.6)	55 (90.2)		3.00 (3.00–5.00)		45 (86.5)	
Immunotherapy ***							
Yes	18 (18.2)	17 (94.4)	0.41	3.00 (2.75–4.00)	0.30	12 (66.7)	0.90
No	81 (81.8)	71 (87.7)		3.00 (3.00–5.00)		58 (71.6)	
Targeted therapy ***							
Yes	44 (44.4)	38 (86.4)	0.48	4.00 (3.00–5.00)	0.07	34 (77.3)	0.29
No	55 (55.6)	50 (90.9)		3.00 (2.00–4.00)		36 (65.5)	
**KPS**							
60–80	36 (36.4)	32 (88.9)	1.00	4.00 (3.00–5.00)	0.54	24 (66.7)	0.50
90–100	63 (63.6)	56 (88.9)		3.00 (2.75–4.00)		45 (73.0)	
**BFI Severity**							
Mild/Moderate (1–6)	63 (63.6)	57 (90.5)	0.86	3.00 (3.00–4.00)	0.54	44 (79.8)	0.22
Severe (≥7)	28 (28.3)	25 (89.3)		3.50 (3.00–5.00)		23 (82.1)	
**HADS-Anxiety**							
No (HADS < 8)	44 (44.4)	40 (90.9)	0.97	3.00 (3.00–5.00)	0.77	31 (70.5)	0.59
Yes (HADS ≥ 8)	45 (45.5)	41 (91.1)		3.00 (3.00–4.00)		34 (75.6)	
**HADS-Depression**							
No (HADS < 8)	59 (59.6)	52 (88.1)	0.39	3.00 (3.00–5.00)	0.39	43 (72.9)	0.82
Yes (HADS ≥ 8)	32 (32.3)	30 (93.8)		3.00 (3.00–4.00)		24 (75.0)	
**ESAS**							
Mild (ESAS 10-<40)	67 (67.7)	60 (89.6)	0.69	3.00 (3.00–4.00)	0.83	47 (70.1)	0.51
Moderate or severe (ESAS ≥ 40–90)	26 (26.3)	24 (92.3)		3.00 (3.00–4.00)		20 (76.9)	
**ISI**							
No/mild (ISI 0–14)	65 (65.7)	64 (98.5)	0.99	3.00 (3.00–4.00)	0.13	46 (70.8)	0.33
Moderate or severe (ISI ≥ 15)	26 (26.3)	18 (27.7)		4.00 (3.00–5.00)		21 (80.8)	

Not all cell values add up due to missing data. ^1^ *Registered—Yes* indicates registration for at least one class (≥1 vs. 0 classes) among all participants (*N* = 99). ^2^ *Number of Classes Registered* (median, IQR) was calculated only among participants who registered for at least one class (*N* = 88). ^3^ *Registered for 3 or More Classes* compares participants who registered for ≥3 classes versus those who registered for 0–2 classes including non-registrants, among all participants (*N* = 99). ^a^ 35 of 47 patients receiving radiation were breast cancer patients. * *p*-values were based on Chi-square tests; *p*-value < 0.05 is statistically significant. ** *p*-values were based on Wilcoxon rank-sum test or Kruskal–Wallis test; *p*-value < 0.05 is statistically significant. *** Patients could receive multiple forms of therapy.

**Table 2:** Class Type Registration by Sociodemographic and Clinical Characteristics.

	Total (*N* = 99)	Fitness		Yoga		Meditation		Tai Chi/ Qigong		Music Therapy		Dance	
		(*N* = 63)		(*N* = 64)		(*N* = 49)		(*N* = 47)		(*N* = 17)		(*N* = 14)	
	*N* (%)	*N* (%)	*p*-Value *	*N* (%)	*p*-Value *	*N* (%)	*p*-Value *	*N* (%)	*p*-Value *	*N* (%)	*p*-Value *	*N* (%)	*p*-Value *
**Gender**			0.30		0.50		0.46		0.47		0.18		1.00
Female	91 (91.9)	56 (61.5)		59 (64.8)		46 (50.5)		44 (48.4)		14 (15.4)		13 (14.3)	
Male	8 (8.1)	7 (87.5)		5 (62.5)		3 (37.5)		3 (37.5)		3 (37.5)		1 (12.5)	
**Age (years)**			0.47		0.16		0.86		0.10		**0.01**		0.48
<65	57 (57.6)	35 (61.4)		40 (70.2)		28 (49.1)		31 (54.4)		5 (8.8)		7 (12.3)	
≥65	42 (42.4)	28 (66.7)		24 (57.1)		21 (50.0)		16 (38.1)		12 (28.6)		7 (16.7)	
**Race**			0.87		0.22		0.13		0.57		1.00		1.00
White	78 (78.8)	52 (66.7)		55 (70.5)		38 (48.7)		38 (48.7)		14 (17.9)		12 (15.4)	
Non-White	21 (21.2)	11 (52.4)		9 (42.9)		11 (52.4)		9 (42.9)		3 (14.3)		2 (9.5)	
**Ethnicity**			0.30		0.50		1.00		**0.04**		1.00		0.61
Non-Hispanic or Latino	88 (88.9)	56 (63.6)		59 (67.0)		45 (51.1)		40 (45.5)		16 (18.2)		12 (13.6)	
Other	11 (11.1)	7 (63.6)		5 (45.5)		4 (36.4)		7 (63.6)		1 (9.1)		2 (18.2)	
**Marital Status**			0.14		0.74		0.75		0.49		0.10		0.33
Married, living w/partner	62 (62.6)	44 (71.0)		40 (64.5)		32 (51.6)		32 (51.6)		9 (14.5)		11 (17.7)	
Single/divorced/separated/widowed	27 (27.3)	13 (48.1)		16 (59.3)		13 (48.1)		14 (51.9)		7 (25.9)		2 (7.4)	
**Education**			0.20		0.09		0.52		0.43		1.00		0.50
High school or some college	20 (20.2)	12 (60.0)		11 (55.0)		10 (50.0)		13 (65.0)		4 (20.0)		2 (10.0)	
College degree or higher	69 (69.7)	45 (65.2)		45 (65.2)		35 (50.7)		33 (47.8)		12 (17.4)		11 (15.9)	
**Employment**			0.22		**0.02**		0.50		0.65		1.00		**0.01**
Employed	46 (46.5)	26 (56.5)		33 (71.7)		24 (52.2)		22 (47.8)		8 (17.4)		2 (4.3)	
Not employed	43 (43.4)	31 (72.1)		23 (53.5)		21 (48.8)		24 (55.8)		8 (18.6)		11 (25.6)	
**Cancer Basket**			0.09		0.68		0.86		0.25		**0.04**		0.39
Breast	35 (35.4)	20 (57.1)		20 (57.1)		14 (40.0)		15 (42.9)		6 (17.1)		5 (14.3)	
Gyn	22 (22.2)	18 (81.8)		15 (68.2)		12 (54.5)		11 (50.0)		2 (9.1)		3 (13.6)	
Head/Neck	13 (13.1)	7 (53.8)		7 (53.8)		6 (46.2)		6 (46.2)		3 (23.1)		1 (7.7)	
Thoracic	25 (25.3)	17 (68.0)		18 (72.0)		15 (60.0)		15 (60.0)		3 (12.0)		3 (12.0)	
Melanoma	4 (4.0)	1 (25.0)		4 (100.0)		2 (50.0)		0 (0.0)		3 (75.0)		2 (50.0)	
**Cancer Stage**			0.46		0.36		0.89		0.99		0.40		**0.03**
Stage 0-III	67 (67.7)	43 (64.2)		44 (65.7)		32 (47.8)		31 (46.3)		13 (19.4)		13 (19.4)	
Stage IV	32 (32.3)	20 (62.5)		20 (62.5)		17 (53.1)		16 (50.0)		4 (12.5)		1 (3.1)	
Cancer Treatment Modality **													
Radiation			0.92		0.43		0.62		0.58		0.85		0.77
Yes	47 (47.5)	27 (57.4)		26 (55.3)		20 (42.5)		19 (40.4)		7 (14.9)		5 (10.6)	
No	52 (52.5)	36 (69.2)		38 (73.1)		29 (55.8)		28 (53.8)		10 (19.2)		9 (17.3)	
Chemotherapy			0.96		0.89		0.33		0.40		0.27		0.93
Yes	37 (37.4)	23 (62.2)		23 (62.2)		20 (54.0)		19 (51.3)		4 (10.8)		5 (13.5)	
No	62 (62.6)	40 (64.5)		41 (66.1)		29 (46.8)		28 (45.2)		13 (21.0)		9 (14.5)	
Immunotherapy			0.48		0.41		0.40		0.26		0.18		1.00
Yes	18 (18.2)	11 (61.1)		11 (61.1)		11 (61.1)		7 (38.9)		1 (5.6)		3 (16.7)	
No	81 (81.8)	52 (64.2)		53 (65.4)		58 (71.6)		40 (49.4)		16 (19.8)		11 (13.6)	
Targeted therapy			0.97		0.43		0.90		0.62		0.06		0.67
Yes	44 (44.4)	28 (63.6)		30 (68.2)		22 (50.0)		22 (50.0)		11 (25.0)		7 (15.9)	
No	55 (55.6)	35 (63.6)		34 (61.2)		27 (49.1)		25 (45.5)		6 (10.9)		7 (12.7)	
**KPS**			0.96		0.39		0.94		0.97		0.27		1.00
60–80	36 (36.4)	23 (63.9)		25 (69.4)		18 (50.0)		17 (47.2)		4 (11.1)		5 (13.9)	
90–100	63 (63.6)	40 (63.5)		39 (61.9)		31 (49.2)		30 (47.6)		13 (20.6)		9 (14.3)	
**BFI Severity**			0.87		0.22		0.64		0.34		0.63		0.75
Mild/Moderate (1–6)	63 (63.6)	40 (63.5)		38 (60.3)		31 (45.6)		30 (47.6)		11 (17.5)		10 (15.9)	
Severe (≥7)	28 (28.3)	18 (64.3)		20 (71.4)		15 (53.6)		16 (57.1)		6 (21.4)		3 (10.7)	
**HADS-Anxiety**			0.26		0.92		0.35		0.38		0.50		0.29
No (HADS < 8)	44 (44.4)	25 (56.8)		28 (63.6)		24 (54.5)		20 (45.5)		9 (20.5)		8 (18.2)	
Yes (HADS ≥ 8)	45 (45.5)	31 (68.9)		29 (64.4)		21 (46.7)		25 (55.6)		7 (15.6)		5 (11.1)	
**HADS-Depression**			0.11		0.54		0.59		0.19		0.49		0.76
No (HADS < 8)	59 (59.6)	40 (67.8)		38 (64.4)		28 (47.5)		32 (54.2)		12 (20.3)		9 (15.3)	
Yes (HADS ≥ 8)	32 (32.3)	18 (56.3)		20 (62.5)		18 (56.3)		14 (43.8)		5 (15.6)		4 (12.5)	
**ESAS**			0.94		0.13		0.45		0.95		0.20		0.33
Mild (ESAS 10-<40)	67 (67.7)	42 (62.7)		40 (59.7)		32 (47.8)		33 (49.3)		10 (14.9)		11 (16.4)	
Moderate or severe (ESAS ≥ 40–90)	26 (26.3)	17 (65.4)		20 (76.9)		15 (57.7)		13 (50.0)		7 (26.9)		2 (7.7)	
**ISI**			0.99		0.28		0.22		0.45		0.99		0.75
No/mild (ISI 0–14)	65 (65.7)	41 (63.1)		39 (86.7)		30 (46.2)		31 (47.7)		12 (18.5)		10 (15.4)	
Moderate or severe (ISI ≥ 15)	26 (26.3)	17 (65.4)		19 (73.1)		16 (61.5)		15 (57.7)		5 (19.2)		3 (11.5)	

Not all cell values add up due to missing data. * *p*-values were based on Chi-square or Fisher’s exact tests as appropriate based on expected cell counts, *p*-value < 0.05 is statistically significant. ** Patients could receive multiple forms of therapy.

## Data Availability

Original data will be provided by the corresponding author upon reasonable request.

## Abbreviations

The following abbreviations are used in this manuscript:

IM@Home	Integrative Medicine at Home
PRO	Patient-Reported Outcomes
RCT	Randomized Clinical Trial
EUC	Enhanced usual care
OIFS	One-Item Fatigue Screen
KPS	Karnofsky Performance Status
BFI	Brief Fatigue Inventory
HADS	Hospital Anxiety and Depression Scale
ESAS	Edmonton Symptom Assessment System
ISI	Insomnia Severity Index
IQR	Interquartile ranges
EHR	Electronic Health Record
MSK	Memorial Sloan Kettering Cancer Center
IRB	Institutional Review Board
